# Fatigue and Its Association with Upper Limb Function in People with Multiple Sclerosis

**DOI:** 10.3390/neurolint17060088

**Published:** 2025-06-09

**Authors:** Erica Grange, Davide Marengo, Rachele Di Giovanni, Giampaolo Brichetto, Margit Mueller, Andrea Tacchino, Rita Bertoni, Francesco Zagari, Angelo Pappalardo, Luca Prosperini, Rosalba Rosato, Davide Cattaneo, Claudio Solaro

**Affiliations:** 1 Scientific Research Area, Italian Multiple Sclerosis Foundation (FISM), 16126 Genova, Italy; erica.grange@fismets.it (E.G.);; 2 CRRF “Mons. Luigi Novarese”, 13040 Moncrivello, Italy; 3 Department of Psychology, University of Turin, 10124 Turin, Italy; 4 IRCSS Fondazione Don Carlo Gnocchi, 20148 Milan, Italy; 5 MS Center Institute of Neurological Sciences, University of Catania, 95124 Catania, Italy; 6 Department of Neurology and Psychiatry, Sapienza University, 00185 Rome, Italy; 7 Department of Physiopathology and Transplants, University of Milan, 20122 Milan, Italy; 8 Neurology Unit, Galliera Hospital, 16128 Genova, Italy

**Keywords:** multiple sclerosis, fatigue, upper limb function, perceived manual ability

## Abstract

**Background and Objectives:** This cross-sectional study investigates the association between fatigue and upper limb (UL) function in people with Multiple Sclerosis (PwMS). **Methods:** Adult PwMS were recruited from five Italian MS centers. Fatigue was evaluated using the Modified Fatigue Impact Scale (MFIS), while UL function was assessed through the Box and Block Test (BBT), Nine-Hole Peg Test (9HPT), and Hand-Grip Strength (HGS). Data analysis included Spearman rank correlations and Mann–Whitney U tests. **Results:** A total of 261 participants were involved. Significant correlations were found between fatigue severity, UL function, and patient-reported manual ability. Physical and cognitive aspects of fatigue were independently related to functional impairments. Participants with clinically relevant fatigue demonstrated lower subjective UL function, poorer BBT and HGS performance, and greater HGS asymmetry. **Discussion:** The study underscores the complex relationship between fatigue and functional impairments in MS. The findings suggest both strength and dexterity contribute to the perception of clinically relevant fatigue in PwMS, highlighting the importance of incorporating both domains to complement neurological assessment. **Conclusion:** Fatigue in PwMS is linked to both subjective and objective measures of UL function. Assessing strength and dexterity alongside fatigue may enhance clinical understanding and inform targeted rehabilitation strategies.

## 1. Introduction

Multiple Sclerosis (MS) is a chronic autoimmune demyelinating disease that predominantly affects the central nervous system, leading to a wide range of neurological symptoms and variable levels of disability [1]. Among the many challenges faced by people with MS (PwMS), upper limb (UL) dysfunction is particularly impactful, affecting approximately 50% of PwMS [2]. UL impairment significantly undermines the quality of life, reducing independence in daily activities and community participation. This is further complicated by fatigue, a pervasive and debilitating symptom in MS affecting both physical and cognitive skills necessary for daily living [3,4].

In exploring the literature, we could only find a few studies exploring the associations between perceived fatigue and UL function in PwMS [5,6,7,8]. Early small-sample research by Yozbatiran and colleagues [5] found that while PwMS exhibited greater fatigue and UL dysfunction compared to controls, fatigue was modestly associated with dexterity tests like the Nine-Hole Peg Test (9-HPT) and self-reported disability. A subsequent study by Padua and colleagues [6], conducted on a larger sample of 80 PwMS, reinforced these findings by showing that fatigue correlated more strongly with self-perceived UL disability than with timed motor performance measures, emphasizing the subjective nature of fatigue’s impact. In contrast, in comparing 20 PwMS with a control group of healthy individuals, Severijns and colleagues [7] reported that UL motor fatigability was significantly higher in PwMS and meaningfully associated with both perceived fatigue and perceived arm use in daily life, suggesting that localized objective fatigability may help explain subjective fatigue reports. In turn, recent findings from another research group only showed weak and non-significant correlations between UL function and fatigue, possibly due to limited sample size (n = 11) and statistical power [8]. Together, these mixed findings point to a nuanced and possibly context-dependent relationship between fatigue and UL function in PwMS. However, most prior studies have relied on small samples, a narrow set of motor or patient-reported outcome (PRO) measures, or lacked a comprehensive integration of subjective and objective UL assessments.

This study aims to fill this existing gap and investigate the association between fatigue and UL function in a substantial sample of PwMS considering a wide set of objective UL assessments and PRO measures. In this way, we strive to provide a more extensive understanding of how fatigue impacts daily living and functional abilities in MS.

## 2. Materials and Methods

### 2.1. Procedure

The present cross-sectional study was conducted between January 2016 and August 2019 in five Italian MS centers. Recruited subjects had a confirmed diagnosis of MS [9], age ≥ 18 years, absence of relapses in the previous 3 months or other neurological disease, ability to understand study procedures and provided informed consent. Subjects were administered an assessment protocol including demographic and clinical characteristics, EDSS, the Symbol Digit Modalities Test (SDMT), and additional measures assessing fatigue and UL function. Fatigue was assessed using the Modified Fatigue Impact Scale (MFIS), encompassing total score and sub-scores for physical and cognitive aspects. UL function was assessed using the Box and Block Test (BBT), Nine-Hole Peg Test (9HPT), Hand-Grip Strength (HGS) on both arms. Additionally, the Manual Ability Measure-36 (MAM-36) PRO was administered.

### 2.2. Measures

#### 2.2.1. Subjective Fatigue

The Modified Fatigue Impact Scale (MFIS-21) [10] is a 21-item questionnaire designed to assess the perceived impact of fatigue on daily functioning over the past four weeks. Each item is rated on a 5-point Likert scale ranging from 0 (never) to 4 (almost always), with higher scores indicating greater perceived fatigue. The MFIS-21 yields a total score and three subscale scores: physical (9 items), cognitive (10 items), and psychosocial (2 items). For the purpose of the present study, the total score, the physical fatigue score, and the cognitive fatigue score were used. The scores showed excellent reliability based on Cronbach’s Alpha (Physical: α = 0.93; Cognitive: α = 0.94; Total score: α = 0.95).

#### 2.2.2. Objective Upper Limb Measures

Objective UL function was assessed via the 9HPT, BBT, and HGS measures. The 9-HPT assesses fine manual dexterity [11]. Participants were instructed to pick up nine small pegs from a container, one at a time, and place them into holes on a board as quickly as possible, then remove each peg and return it to the container. For this study, the time (in seconds) to complete each trial was recorded, with a maximum duration of 180 s. Each participant completed two trials per arm, and the final score for each arm was calculated as the average time across both trials. The BBT [12], a measure of gross manual dexterity, involves transferring as many blocks as possible, one at a time, from one compartment of a divided box to the other within 60 s. The score for each arm corresponds to the number of blocks successfully moved within the time limit. HGS, quantifying the maximum isometric strength of each hand, was evaluated with the Jamar^®^ hydraulic dynamometer (Patterson Medical, Warrenville, IL, USA) with the participant seated in a standardized chair (low-backed, fixed armrests), feet flat on the floor. The shoulder was adducted, elbow flexed at 90°, and the forearm and wrist maintained in a neutral position, with the wrist just beyond the end of the armrest and the thumb facing upward. The participant performed three maximal-effort contractions while the examiner stabilized the base of the dynamometer. The grip strength was scored in kilograms, with the final score being the mean of three HGS trials [13]. For all measures inter-arm average and asymmetry (absolute difference) scores were computed.

#### 2.2.3. Subjective Upper Limb Measure

We administered the Italian adaptation of the MAM-36 [14], consisting of 36 items assessing participants’ self-perceived ability to perform everyday upper limb tasks (e.g., eating, dressing, buttoning clothes), without the aid of adaptive equipment. The questionnaire includes 36 items, each rated on a 4-point scale from 1 (cannot do it) to 4 (easy to do). Items referring to tasks that are rarely or never performed are scored as 0. For this study, the total MAM-36 score was calculated by summing the item responses. The measure demonstrated excellent internal consistency, with a Cronbach’s α = 0.96.

### 2.3. Data Analysis

First, the normality of measures was verified using the Shapiro–Wilk test. All measures significantly deviated from normality, except for the average BBT score (*p* = 0.227). For this reason, subsequent analyses were performed using non-parametric approaches. Spearman rank correlations were utilized to analyze the association between fatigue measures (MFIS Total, Physical and Cognitive Fatigues), and subjective (e.g., MAM-36) and objective UL measures (e.g., 9HPT, BBT, HGS), both as inter-arm average, and asymmetry. Finally, we looked at differences in the distribution of subjective and objective UL measures and cognitive function across the two groups reporting MFIS total scores equal to or above the cut-off for clinically relevant fatigue (i.e., MFIS ≥ 38) [15], or those sitting below the cut-off. Differences in the distribution of the scores in the two groups were evaluated using non-parametric Mann–Whitney U test. Between-group differences were visualized using a radar chart plotting the median percentile rank of subjective and objective measures in the two groups. Percentile ranks were used to allow for comparison across upper limb measures with different metrics. Statistical significance for all associations and group comparisons was evaluated at *p* < 0.05 (unadjusted) and after applying the false discovery rate (FDR) correction to control for multiple hypothesis testing. Analyses were performed with IBM SPSS Statistics, version 29.

## 3. Results

### 3.1. Sample Characteristics

A sample of n = 261 PwMS participated in the study (see Table 1 for sample characteristics). The mean age was 51.4 years (SD = 13.3; range = 19–85), and the majority were female (n = 168, 64.4%). The average disease duration was 14.6 years (SD = 10.8), with a broad range from newly diagnosed to over five decades post-diagnosis. Regarding disease course, 52.9% had relapsing-remitting MS (n = 138), 18.4% had primary progressive MS (n = 48), and 28.7% had secondary progressive MS (n = 75). Disability levels, as measured by the Expanded Disability Status Scale (EDSS), showed a median value of 6 (range = 0–8.5), indicating a sample with moderate to severe disability. SDMT had a mean score of 38.0 (SD = 14.0; range = 6–73), with a median of 37.

Fatigue scores (MFIS) showed an average total score of 32.7 (SD = 19.0), and mean subscale scores of 18.2 (SD = 9.7) for physical fatigue and 11.2 (SD = 9.7) for cognitive fatigue. Next, we look at the UL function. Of note, 95.4% of participants were right-handed (n = 249), 4.2% were left-handed (n = 11), and only 0.4% identified as ambidextrous (n = 1). On the BBT, on average participants transferred 44.8 blocks (SD = 14.6), with a mean inter-arm asymmetry of 7.3 blocks (SD = 7.7). Hand-grip strength HGS averaged 19.5 kg (SD = 8.8), with a mean asymmetry of 5.7 kg (SD = 5.9). The average completion time for the Nine-Hole Peg Test (9HPT) was 41.2 s (SD = 32.9), and asymmetry in 9HPT performance averaged 19.2 s (SD = 35.3). Perceived manual ability, assessed through the MAM-36, was relatively high on average, with a mean total score of 123.7 (SD = 20.2), out of a maximum of 144.

### 3.2. Correlation Between Fatigue and Upper Limb Measures

Results of Spearman correlations computed between MFIS scores (total, physical, cognitive) and indices of UL function are reported in Table 2 along with unadjusted and FDR-adjusted p values. Significant negative correlations were found between MFIS Total and both average BBT (rho = −0.189, *p* = 0.002, adj. *p* = 0.005) and average HGS (rho = −0.210, *p* = 0.001, adj. *p* = 0.003); in turn, a positive correlation emerged between the MFIS total and HGS asymmetry (rho = 0.153, *p* = 0.014, adj. *p* = 0.026). No significant associations were observed with either 9HPT average or asymmetry, or with BBT asymmetry.

MFIS Physical scores showed a similar pattern. They correlated negatively with the average BBT score (rho = −0.234, *p* < 0.001, adj. *p* = 0.001), average HGS (rho = −0.192, *p* = 0.002, adj. *p* = 0.005), and positively with both HGS asymmetry (rho = 0.197, *p* = 0.001, adj. *p* = 0.005) and 9HPT asymmetry, although this correlation was no longer significant after FDR correction (rho = 0.135, *p* = 0.029, adj. *p* = 0.051). A significant positive correlation was also found with the average 9HPT score (rho = 0.196, *p* = 0.001, adj. *p* = 0.004). Asymmetry on the BBT showed no significant correlations.

MFIS Cognitive scores were negatively correlated with average HGS (rho = −0.173, *p* = 0.005, adj. *p* = 0.011) and asymmetry on the 9HPT, although this correlation was no longer significant after FDR correction (rho = −0.133, *p* = 0.031, adj. *p* = 0.051). No associations were found between MFIS Cognitive scores and the average BBT scores, BBT asymmetry, HGS asymmetry, or average 9HPT scores.

All MFIS scores showed significant negative correlations with MAM-36: MFIS Total (rho = −0.480, *p* < 0.001, adj. *p* = <0.001), MFIS Physical (rho = −0.515, *p* < 0.001, adj. *p* = <0.001), and MFIS Cognitive (rho = −0.312, *p* < 0.001, adj. *p* = <0.001), indicating a consistent relationship between fatigue and perceived manual ability.

### 3.3. Upper Limb Function by Fatigue Severity

**Figure 1:**
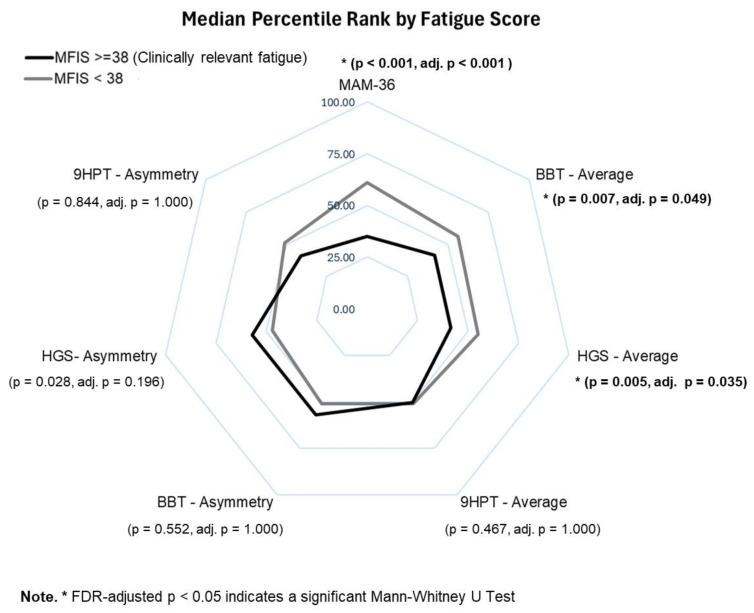
Radar chart plotting median percentile rank of UL measures by MFIS severity, with higher percentile value indicating a higher score.

Participants with clinically significant fatigue (MFIS ≥ 38) reported lower perceived manual ability, with MAM-36 scores significantly lower than those without clinical fatigue (U = 4793.0, *p* < 0.001, adj *p* < 0.001). Regarding objective UL function, the group reporting clinically relevant fatigue showed significantly reduced performance on the BBT (U = 6599.5, *p* = 0.007, adj. *p* = 0.049), weaker HGS (U = 6546.0, *p* = 0.005, adj. *p* = 0.035), and greater asymmetry in HGS, although this group difference was no longer significant after FDR correction (U = 9530.5, *p* = 0.028, adj. *p* = 0.196). In contrast, no significant differences were observed for the 9HPT in terms of average performance or asymmetry, nor for asymmetry on the BBT. The radar chart in Figure 1 highlights the contrast in functional profiles between groups, illustrating more pronounced impairments in the fatigue group across both subjective and objective domains. The figure compares median percentile scores for each UL measure between participants reporting clinically relevant fatigue (MFIS ≥ 38) and those reporting lower levels of fatigue. Combined, these findings appear to suggest that fatigue is more closely related to deficits in strength and gross manual dexterity (as measured by HGS and BBT), rather than to fine motor control (as measured by 9HPT).

## 4. Discussion

The present study demonstrates small-to-moderate significant associations between fatigue severity and various measures of UL function, as well as self-reported manual ability, in PwMS. Consistent with the previous literature, more fatigued subjects showed poorer performances in objective-measure UL motor function [6,7], and overall patient-reported manual ability [16,17]. Of note, a pattern emerged indicating a small but significant association between perceived physical fatigue and performance on all the considered UL measures, which included the 9HPT, BBT and HGS, and perceived manual ability assessed using the MAM-36. Note, however, that clinically relevant fatigue (MFIS ≥ 38) was associated with lower perceived manual ability, and poorer performances on the BBT and HGS, but not on the 9HPT.

Differentiating between physical and cognitive fatigue highlighted that both independently relate to functional impairments and reduced manual ability in PwMS, particularly affecting fine motor coordination. Of note, we observed positive but small correlations between asymmetry in hand-grip strength and dexterity and physical fatigue, possibly reflecting asymmetrical manifestations of MS pathology in motor function. Conversely, higher cognitive fatigue showed a marginally significant association with lower asymmetry on the 9HPT, a small but unexpected finding that might suggest a link between cognitive fatigue and fine motor coordination. Given the complex relationship between mean dexterity performance, asymmetry, and disability [18], we re-examined this association while controlling for average dexterity. Under this condition, the correlation between cognitive fatigue and 9HPT asymmetry was weakened. Overall, due to the exploratory nature of these analyses, the observed associations should be interpreted with caution. Future studies are needed to further investigate these relationships and clarify the underlying mechanisms.

Overall, our findings underscore the importance of incorporating fatigue into the comprehensive assessment and management of multiple sclerosis (MS). They also prompt further consideration of the relative contributions of strength and dexterity to the perception of fatigue in PwMS, reinforcing the need to assess both domains alongside standard neurological evaluations [6,7,19]. At the same time, while fatigue appears to be associated with poorer physical outcomes and reduced perceived manual ability, the correlational nature of the present study limits our ability to determine whether fatigue is a contributing factor to, or a consequence of, these impairments. Longitudinal studies are needed to elucidate the directionality and causal pathways underlying these associations.

This study has strengths that enhance the robustness and clinical relevance of its findings. The use of a large, multicenter sample increases the generalizability of the results across different clinical settings and populations of PwMS. Additionally, the integration of both objective assessments of upper limb function (BBT, 9HPT, HGS) and validated patient-reported outcomes (MFIS, MAM-36) allowed for a comprehensive evaluation of the relationship between fatigue and manual ability. The distinction between physical and cognitive fatigue further adds to the depth of the analysis, offering a more nuanced understanding of their independent associations with functional impairments.

However, several limitations should be acknowledged. As noted above, the cross-sectional design precluded causal inferences, limiting our ability to determine the directionality of the observed associations. The use of convenience sampling may have also introduced selection bias and reduce the generalizability of the findings to the broader MS population. Although we employed validated and widely used tools, they may not fully capture the complexity of UL function in PwMS. Furthermore, cognitive function was assessed solely using the SDMT, and potentially relevant variables, such as mood and sleep quality, were not included in the sample characterization. Notably, in examining the association between upper limb (UL) function and fatigue, we chose to report unadjusted correlations to maintain comparability with previous studies [5,6]. This approach also helped minimize the risk of over-adjustment bias [20], given that several potential covariates, such as disability level, may lie on or near the causal pathway between the examined constructs and could attenuate meaningful associations if statistically controlled [21]. While this decision allowed for more direct comparison with prior research, it limited our ability to isolate the independent contribution of individual variables. Future studies should consider incorporating these covariates into assessment protocols and evaluating their role in the relationship between UL function and fatigue. Lastly, the absence of a healthy control group limited our capacity to determine the specificity of our findings to individuals with MS [5]. Future studies could benefit from including a broader range of clinical and psychosocial variables, incorporating healthy comparison groups, and adopting longitudinal designs to build on these findings and further clarify the relationships among fatigue, UL function, and quality of life in PwMS.

## 5. Conclusions

To conclude, our findings indicate a modest association between patient-reported fatigue and objective UL function, and a stronger association with patient-reported manual ability in people with PwMS. Further research is warranted to investigate the mechanisms underlying the observed relationship between fatigue severity and UL function.

## Figures and Tables

**Table 1:** Sample characteristics.

	Mean/N	Median	SD	Min	Max
Sex (Female)	168	-	-	-	-
Age (years)	51.44	52.34	13.30	19.00	85.03
Disease Duration (years)	14.61	12.00	10.84	0.00	53.00
Disease Course (Relapsing Remitting)	138	-	-	-	-
Disease Course (Primary Progressive)	48	-	-	-	-
Disease Course (Secondary Progressive)	75	-	-	-	-
EDSS	5.03	6.00	1.90	0.00	8.50
SDMT	38.03	37.00	14.03	6.00	73.00
MFIS—Total	32.65	30.00	19.01	0.00	82.00
MFIS—Physical	18.15	19.00	9.67	0.00	36.00
MFIS—Cognitive	11.18	9.00	9.71	0.00	38.00
BBT (blocks)—Average	44.84	45.00	14.58	9.50	101.00
BBT (blocks)—Asymmetry	7.28	5.00	7.66	0.00	55.00
HGS (kg)—Average	19.45	18.50	8.76	2.91	50.67
HGS (kg)—Asymmetry	5.73	3.85	5.91	0.00	39.00
9HPT (s)—Average	41.15	29.00	32.86	13.48	223.00
9HPT (s)—Asymmetry	19.23	4.00	35.26	0.00	172.00
MAM-36 TOT	123.72	129.00	20.21	51.00	144.00

**Table 2:** Correlation between fatigue (MFIS) and UL and cognitive measures.

	MFIS Total		MFIS—Physical		MFIS—Cognitive	
	rho	*p* (adj. *p*)	rho	*p*	rho	*p*
BBT (blocks)—Average	−0.189	0.002 (0.005)	−0.234	<0.001 (0.001)	−0.069	0.269 (0.314)
BBT (blocks)—Asymmetry	0.081	0.191 (0.251)	0.096	0.120 (0.168)	0.050	0.419 (0.440)
HGS (kg)—Average	−0.210	0.001 (0.003)	−0.192	0.002 (0.005)	−0.173	0.005 (0.011)
HGS (kg)—Asymmetry	0.153	0.014 (0.026)	0.197	0.001 (0.005)	0.068	0.271 (0.300)
9HPT (s)—Average	0.100	0.107 (0.160)	0.196	0.001 (0.004)	−0.072	0.244 (0.301)
9HPT (s)—Asymmetry	0.027	0.661 (0.661)	0.135	0.029 (0.051)	−0.133	0.031 (0.051)
MAM-36 TOT	−0.480	<0.001 (<0.001)	−0.515	<0.001 (<0.001)	−0.312	<0.001 (<0.001)

## Data Availability

The data is not publicly available due to privacy or ethical restrictions.
